# Supplementary material for: Adenosine integrates light and sleep signalling for the regulation of circadian timing in mice
Source: Nat Commun. 2021 Apr 9;12:2113. doi: 10.1038/s41467-021-22179-z (PMC8035342; doi:10.1038/s41467-021-22179-z)

Supplementary Material

Jagannath et al.

Adenosine integrates light and sleep signalling for the regulation of circadian timing in mice

Supplementary Fig 1

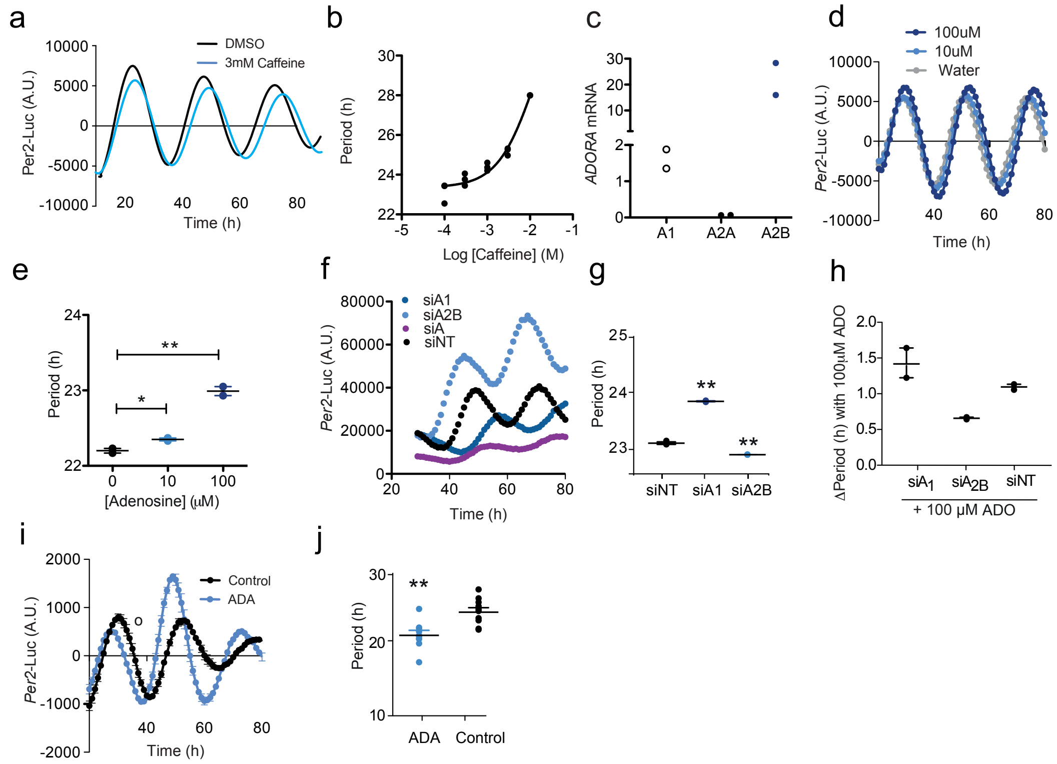

Supplementary Figure 1: Caffeine and adenosine regulate cellular circadian rhythms. (a) Caffeine increased period length in *Per2*-Luc U2OS cells in a concentration-dependent manner, and summarised in (b, n=3); (c) Levels of adenosine receptor subtype (*ADORA*) mRNA in U2OS cells as measured by qPCR, n=2; (d) Period length of *Per2*-Luc U2OS cells treated with 100μM (dark blue), 10μM (light blue) adenosine, and water (grey) and summarised in (e) p=.0016, one-way ANOVA); (f) Representative traces (single) of Per2-Luc U2OS period length wtih siRNA-mediated knockdown of *ADORA1* (siA1, dark blue) and *ADORA2B* (siA2B, light blue), control (siNT, black), with knockdown of both A_1 and A_{2B} in purple (siA). Results (f) are summarised in (g), n=3, p<0 .0000001); (h) Knockdown of A_{2B} receptors reduces the period lengthening effects of adenosine, whilst the knockdown of A_1 produces the opposite effect; (i) Reducing extracellular adenosine by addition of adenosine deaminase (ADA – 0.5U) reduces period length, the control is heat denatured ADA, with results summarised in (j), n=10. (*=p0.0031, two tailed unpaired t-test). Error bars are SEM dot plots.

Supplementary Fig. 2

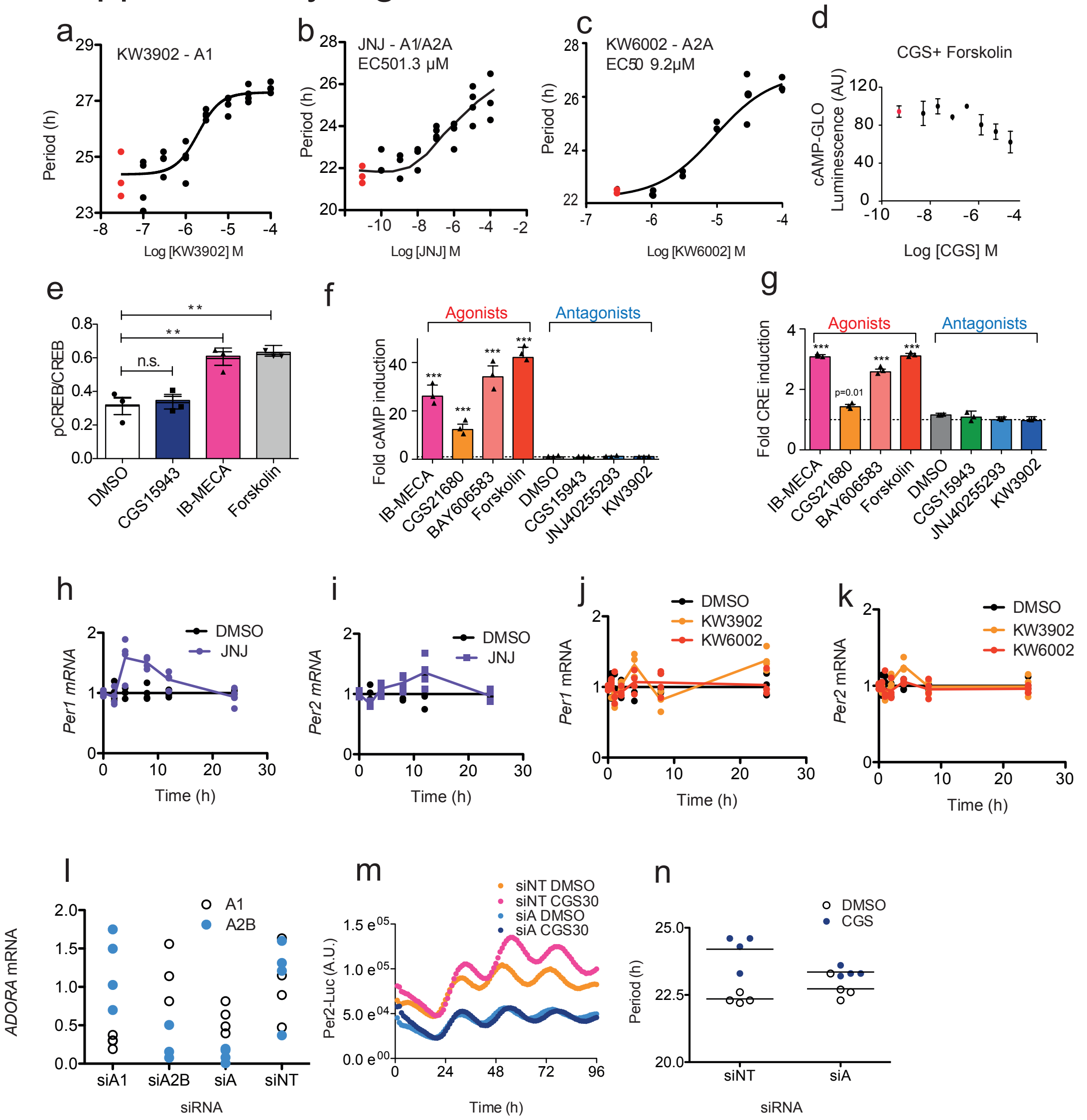

Supplementary Figure 2: Mechanism of action of adenosine signalling to the clock. Concentration-dependent period lengthening in *Per2*-Luc U2OS cells elicited by (a) KW3902 (A_1 antagonist); (b) JNJ (A_{2A}/A_1 antagonist); and (c) KW6002 (A_{2A} antagonist); (d) CGS dose-dependently blocks forskolin (10µM)-mediated increases in cAMP as measured with the cAMP-GloSensor reporter; DMSO controls for a-d indicated in red. (e) Quantification of pCREB from western blots shown in Figure 1 (d) and (I (n=3 p=0.002 and 0.001 for IB-MECA and Forskolin with Dunnes multiple comparison test following one way ANOVA); (f) cAMP increases as monitored by the cAMP-GLO assay and quantified at 15min following drug addition (10 µM), n=3, *** = significantly different from DMSO control, $p < 0.00001$, not quantified or otherwise indicated, one-way ANOVA with Dunnett's post-hoc correction; (g) CRE-Luc induction after 4h of treatment drugs at 10 µM. Dotted lines indicate control levels

and n=3, *** = significantly different from DMSO control, p < 0.00001, not quantified or otherwise indicated, one-way ANOVA with Dunnett's post-hoc correction; (h) JNJ increased *PER1* expression (significant at 4h & 8h, p<0.01) and (i) *PER2* significant at 4h, 8h and 12h (p<0.01); (j and k) In contrast to JNJ, specific A1 and A_{2A} antagonists KW3902 10µM and KW6002 10µM caused no significant changes to *PER1/2* levels; (l) After siRNA-mediated knockdown of *ADORA1* (siA1) *ADORA2B* (siA2B) or both receptors (siA), period lengthening in Per2-Luc U2OS cells by CGS is lost (m) (negative control siNT DMSO, negative control siRNA drug treated - siNT CGS 30µM – C30 Adenosine receptor siRNA DMSO treated - siA DMSO Adenosine receptor siRNA, drug treated - siA CGS 30µM – C30). The results are quantified in (n). Error bars = S.E.M., Two-way ANOVA, *=p<0.05, **=p<0.01 from Bonferroni post-hoc tests.

Supplementary Fig. 3

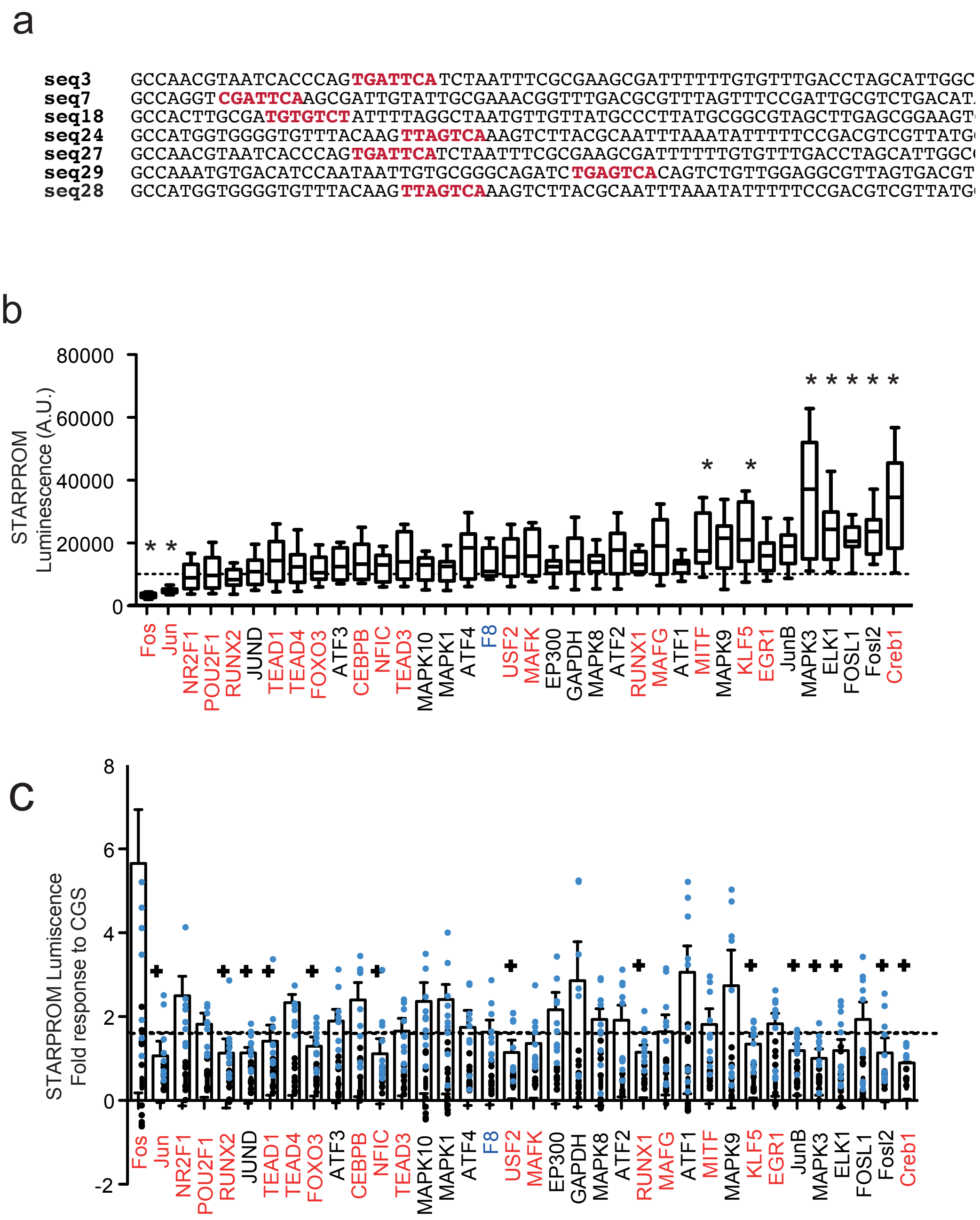

Supplementary Figure 3: STAR-PROM identified the AP-1 response element (RE) downstream of adenosine signalling. (a) Sequence of the 7 clones with AP-1 RE highlighted in red; (b) Baseline expression of clone3 and (c) fold change in response to 10μM CGS. Treatments on x-axis in both (b) and (c) are siRNAs against the genes encoding multiple transcription factors and related signalling elements that were immunoprecipitated by the bait sequence from clone3. Genes whose knockdown significantly altered the expression of clone3 or reduced its response to CGS are indicated in red. Two-way ANOVA with Dunnett's post-

hoc test, $p<0.05$ = * difference from F8 control for (b) and no significant induction in response to CGS indicated by + (c). Dotted lines are negative control siRNA transfected cells and n=8 replicates for this experiment, Tukey's box plots used throughout (central line - mean, box represents 25th to 75th percentile data, whiskers are 1.5 interquartile range). Individual p-values are too many to list, and hence please refer to source data.

Supplementary Fig. 4

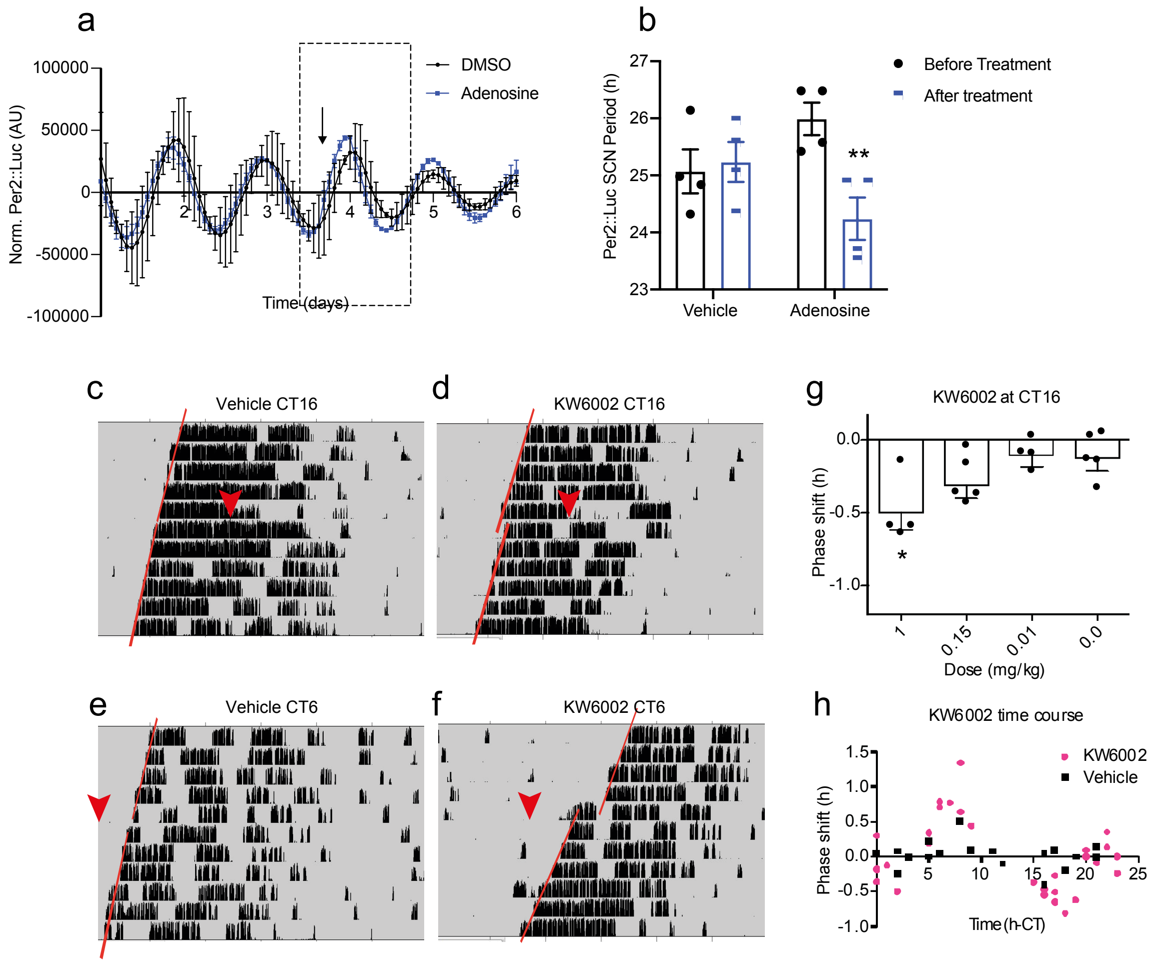

Supplementary Figure 4: A_{2A}/A_1 adenosine receptor antagonists modify circadian wheel running behaviour. (a) Per2::Luc SCN slices treated with 250uM adenosine, added at arrow (n=4), summarised in (b), n=4, p=0.004 after treatment with adenosine which reduces period by 1.7h, no significant difference with vehicle, box indicates analysis window. (c) Animals were housed in constant dark and at the red arrow received either vehicle or (d) 1mg/kg KW6002 (A_{2A} antagonist) at CT16 (c,d) and CT6 (e,f). Onsets of activity were measured (red lines); (g)

phase delay shifts were plotted. KW6002 causes phase delays in a dose-dependent manner at CT16 (n=4-5, p=0.02 for 1mg kw6002 over control with dunnetts multiple comparison, one-way ANOVA) (h) The phase response curve (PRC) to KW6002 (1mg/kg) is plotted and KW6002 induced larger phase shifts than the control. A total of 12 animals received one injection a week for a total of 6 weeks of either KW6002 or vehicle in a randomised manner and data collated. Two-way ANOVA with Bonferroni post-hoc test - *=p<0.05, **=p<0.01. Error bars are SEM on bar charts.

Supplementary Fig. 5

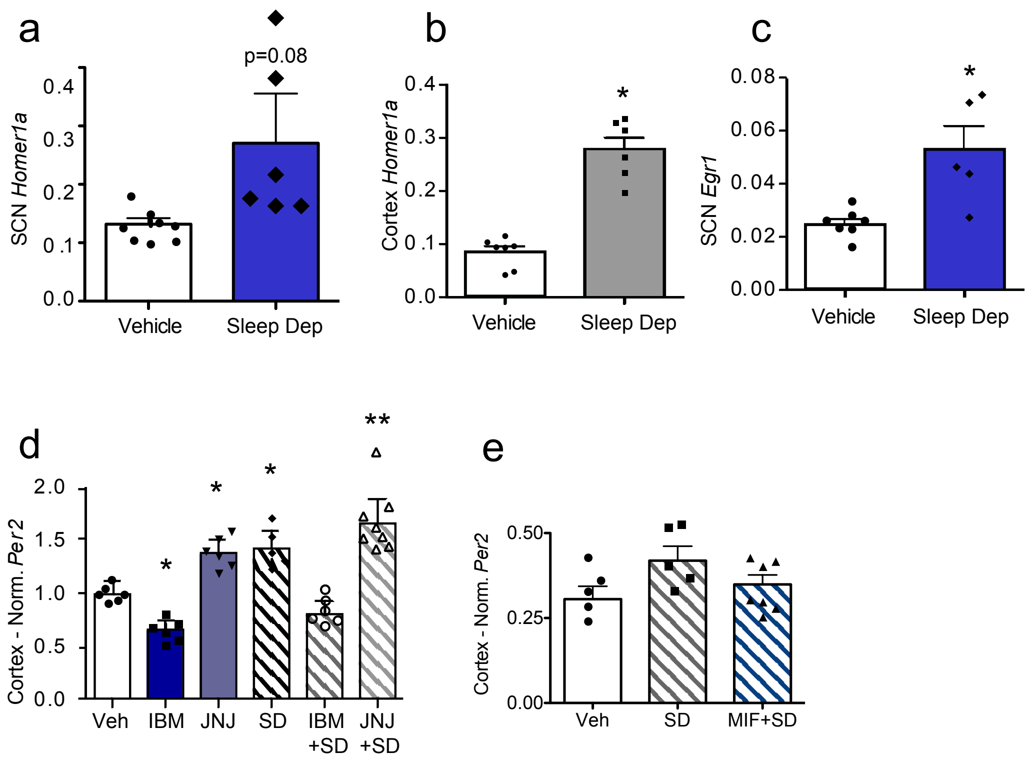

Supplementary Figure 5: Sleep deprivation alters clock gene expression within the SCN and cortex (a) *Homer1a* mRNA levels within the SCN (b) within the cortex immediately after 6h sleep deprivation (c) *Egr1* within the SCN after sleep deprivation. (p=.08, 0.000003303 and .0037 for a,b, and c, two tailed unpaired t-test, n=5-8.). The expression of both genes have previously been shown to be correlated with sleep deprivation[45]. (d) Sleep deprivation increases *Per2* expression within the cortex as previously reported. This effect is enganced by JNJ and supressed by IB-MECA (p=0.0008, SD vs IBM+SD). One-way ANOVA with Sidak's test for multiple comparisons, stars indicate p-values compared with vehicle (p=0.014 for IBM, 0.033 for JNJ, 0.049 for SD, <0.0001 for JNJ+SD). (e) We speculated this increase in *Per2* was as a result of increased glucocorticoid signalling due to sleep deprivation[62]. This was tested by the administration of mifepristone (a glucocorticoid antagonist) which as

anticipated abolished the increase in *Per2*. (n=8, error bars = S.E.M.). Error bars are SEM on bar charts.

Supplementary Fig. 6

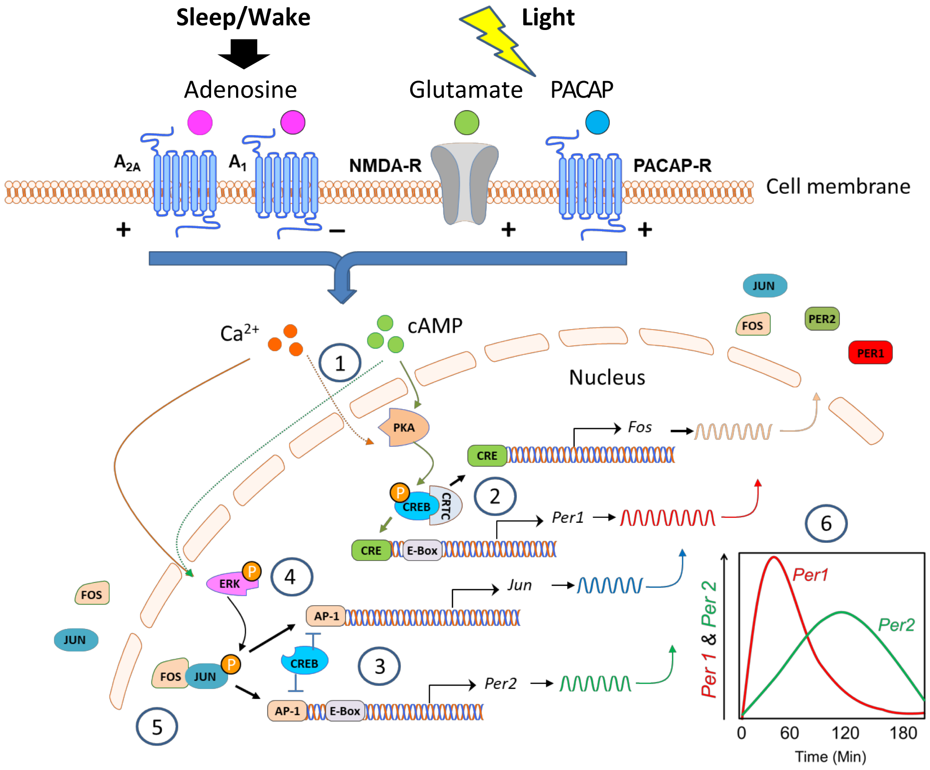

Supplementary Figure 6: Model delineating signalling pathways downstream of both adenosine and light signalling within the SCN. (1) Adenosine signals through G_i (A_1) or G_s (A_{2A}) coupled receptors (Suppl. Fig. 1c- and Fig. 3a) to alter levels of cAMP and intracellular Ca^{2+} [20,21]. As demonstrated in this paper, adenosine receptor (AR) antagonists and agonists differentially alter Ca^{2+} (Fig.2h) and cAMP (Suppl. Fig. 2f) respectively. The adenosine receptor sub-type expressed on a cell will define the response to adenosine. In SCN neurones, the A_1 receptor expression is 5-fold greater than A_{2A} (Fig. 3a). As a result, adenosine will inhibit this pathway. Significantly, adenosine acts upon the same pathways as light. Light induces the release of glutamate and PACAP from photosensitive retinal ganglion cells. NMDA and PACAP receptors activated on SCN neurons release Ca^{2+} and cAMP respectively; **(2)** cAMP activates protein kinase A (PKA) which phosphorylates CREB (Fig. 1d and 3d). pCREB in concert with co-activators such as CRTC binds CREs on *Fos* and *Per1* driving their transcription[2,56]. **(3)** Unphosphorylated CREB competes for AP-1 response elements (REs) resulting in repression[30]. The phosphorylation of CREB (or its removal as in Suppl. Figure 3b-c) will derepress AP-1 REs, including those on *Per2* and *Jun*[30]. **(4)** Increased intracellular Ca^{2+}

results in the activation (phosphorylation) of ERK1/2 (Fig. 2d and Fig. 3d) which increases transcription of *Jun*[24] (Fig. 2g). ERK also phosphorylates JUN, pJUN and FOS heterodimerise to form the AP-1 transcription factor[24]. **(5)** AP-1 drives transcription of *Per2* (Fig. 1j and Fig. 3e), *Jun* (Fig. 2g) and other genes with AP-1 REs (Fig. 2b). **(6)** The net result is a rapid induction of *Per1* through CREB and a slower more sustained induction of *Per2* through AP-1. The resulting phase shift of the circadian clock is therefore the integrated product of sleep/wake history and light.

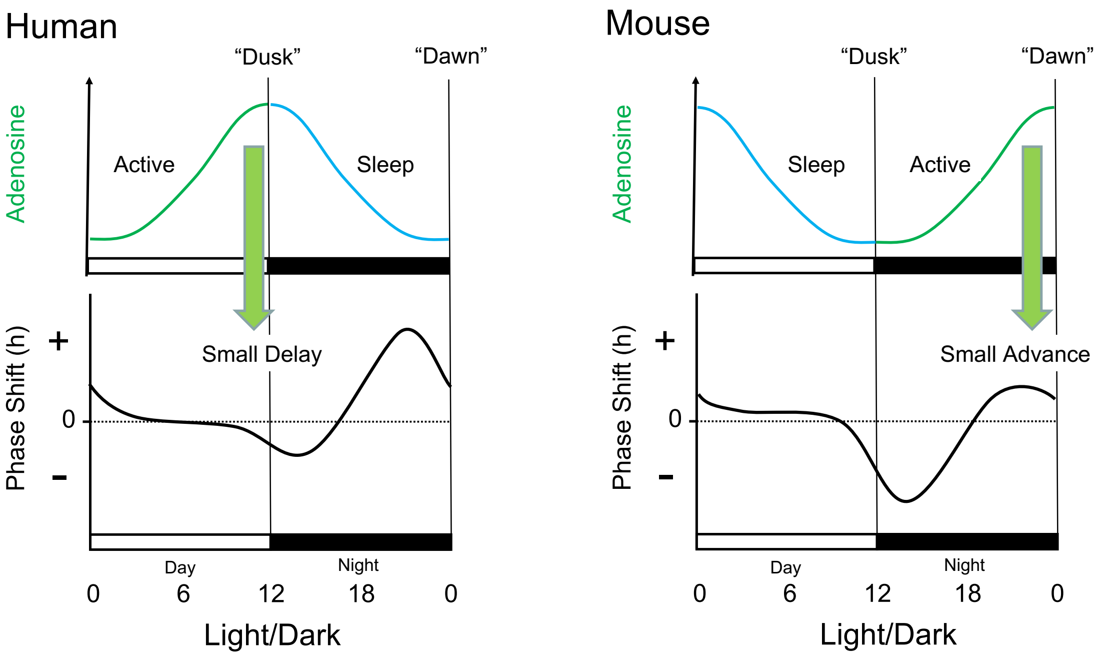

Supplementary Figure 7: Model of adenosine's effect on the phase response curve. In mice, a nocturnal species with a short free-running period (Tau), the PRC shows large delays around dusk and small advances around dawn, whilst the PRC of humans, a diurnal species with long Tau has small delays at dusk and large advances at dawn[63]. Mice would have high levels of adenosine at dawn which would act to attenuate light responses at this time, whereas the reverse would be true at dusk.

Summary table of drugs used and their affinities to the adenosine receptors. Suppl. Table 1.

Name	A1 (Kd/Ex50) nm	A2a (Kd/Ex50) nm	A2B (Kd/Ex50) nm	A3 (Kd/Ex50) nm	A1 vs A2A Selectivity

KW3902	0.19	170	52	>10000	895 (A2a/A1)
KW6002	11,169	151	2,701	1,939	74 (A1/A2a)
CGS15943	3.5	.8	50	>10000	4 (A1/A2a)
JNJ40255293	48	6.6	230	9200	7 (A1/A2a)
IB-Meca	51	2520	11000	1.2	56 (A2a/A1)
Adenosine	100	310	15000	290	3(A2a/A1)

References for drugs:
KW-3902[11]
KW-6002[12]
CGS15943[13]
JNJ-40255293[14]
IB-meca[15]
Adenosine[16]

1 Yoo, S. H. *et al.* PERIOD2::LUCIFERASE real-time reporting of circadian dynamics reveals persistent circadian oscillations in mouse peripheral tissues. *Proc Natl Acad Sci U S A* **101**, 5339-5346, doi:10.1073/pnas.0308709101 (2004).

2 Fedele, D. E. *et al.* Astrogliosis in epilepsy leads to overexpression of adenosine kinase, resulting in seizure aggravation. *Brain* **128**, 2383-2395, doi:10.1093/brain/awh555 (2005).

3 Ramanathan, C. *et al.* Cell type-specific functions of period genes revealed by novel adipocyte and hepatocyte circadian clock models. *PLoS Genet* **10**, e1004244, doi:10.1371/journal.pgen.1004244 (2014).

4 Palchykova, S. *et al.* Manipulation of adenosine kinase affects sleep regulation in mice. *J Neurosci* **30**, 13157-13165, doi:10.1523/JNEUROSCI.1359-10.2010 (2010).

5 Peirson, S. N., Butler, J. N. & Foster, R. G. Experimental validation of novel and conventional approaches to quantitative real-time PCR data analysis. *Nucleic Acids Res* **31**, e73 (2003).

6 Jagannath, A. *et al.* The CRTC1-SIK1 pathway regulates entrainment of the circadian clock. *Cell* **154**, 1100-1111, doi:S0092-8674(13)00961-6 [pii]
10.1016/j.cell.2013.08.004 (2013).

7 Gosselin, P., Rando, G., Fleury-Olela, F. & Schibler, U. Unbiased identification of signal-activated transcription factors by barcoded synthetic tandem repeat promoter screening (BC-STAR-PROM). *Gene Dev* **30**, 1895-1907, doi:10.1101/gad.284828 (2016).

8 Lavery, D. J. & Schibler, U. Circadian Transcription of the Cholesterol 7-Alpha Hydroxylase Gene May Involve the Liver-Enriched Bzip Protein Dbp. *Gene Dev* **7**, 1871-1884, doi:DOI 10.1101/gad.7.10.1871 (1993).

9 Jutras, B. L., Verma, A. & Stevenson, B. Identification of novel DNA-binding proteins using DNA-affinity chromatography/pull down. *Curr Protoc Microbiol* **Chapter 1**, Unit1F 1, doi:10.1002/9780471729259.mc01f01s24 (2012).

10 Masino, S. A., Latini, S., Bordoni, F., Pedata, F. & Dunwiddie, T. V. Changes in hippocampal adenosine efflux, ATP levels, and synaptic transmission induced by increased temperature. *Synapse* **41**, 58-64, doi:10.1002/syn.1060 (2001).

11 Nonaka, H. *et al.* KW-3902, a selective high affinity antagonist for adenosine A1 receptors. *Br J Pharmacol* **117**, 1645-1652, doi:10.1111/j.1476-5381.1996.tb15335.x (1996).

12 Yang, M. *et al.* Characterization of the potency, selectivity, and pharmacokinetic profile for six adenosine A2A receptor antagonists. *Naunyn Schmiedebergs Arch Pharmacol* **375**, 133-144, doi:10.1007/s00210-007-0135-0 (2007).

13 Ongini, E., Dionisotti, S., Gessi, S., Irenius, E. & Fredholm, B. B. Comparison of CGS 15943, ZM 241385 and SCH 58261 as antagonists at human adenosine receptors. *Naunyn Schmiedebergs Arch Pharmacol* **359**, 7-10 (1999).

14 Atack, J. R. *et al.* JNJ-40255293, a novel adenosine A2A/A1 antagonist with efficacy in preclinical models of Parkinson's disease. *ACS Chem Neurosci* **5**, 1005-1019, doi:10.1021/cn5001606 (2014).

15 Klotz, K. N. *et al.* Comparative pharmacology of human adenosine receptor subtypes - characterization of stably transfected receptors in CHO cells. *Naunyn Schmiedebergs Arch Pharmacol* **357**, 1-9 (1998).

16 Yan, L., Burbiel, J. C., Maass, A. & Muller, C. E. Adenosine receptor agonists: from basic medicinal chemistry to clinical development. *Expert Opin Emerg Drugs* **8**, 537-576, doi:10.1517/14728214.8.2.537 (2003).

Fig. 1d and 1i
CREB

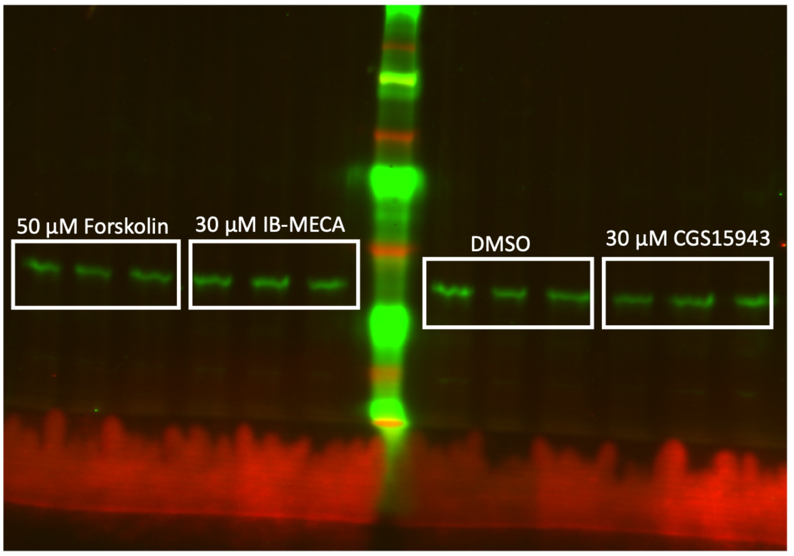

Fig. 1d and 1i
pCREB

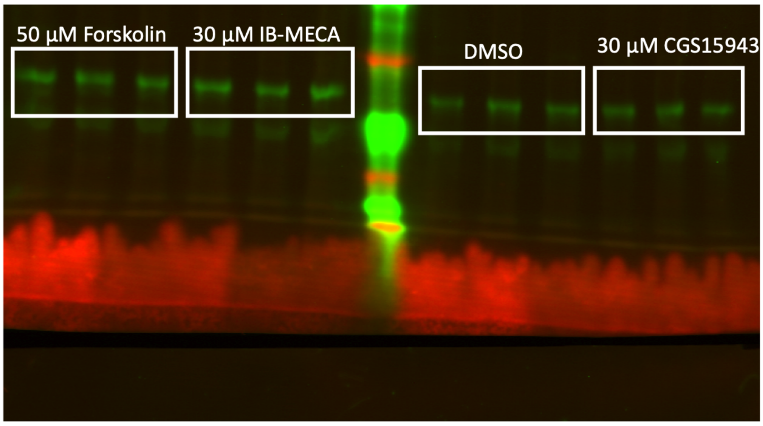

Fig 2f ERK (above) and pERK (below)

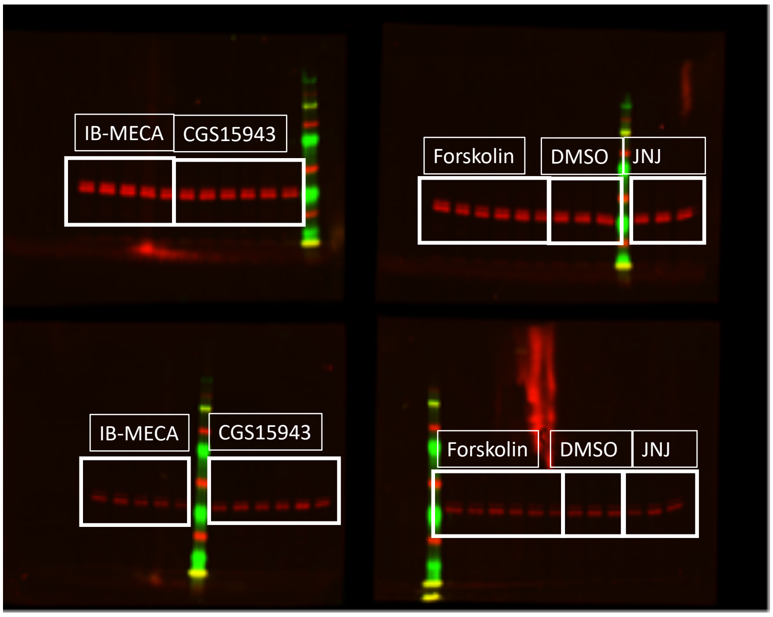

Fig. 3d (pERK)

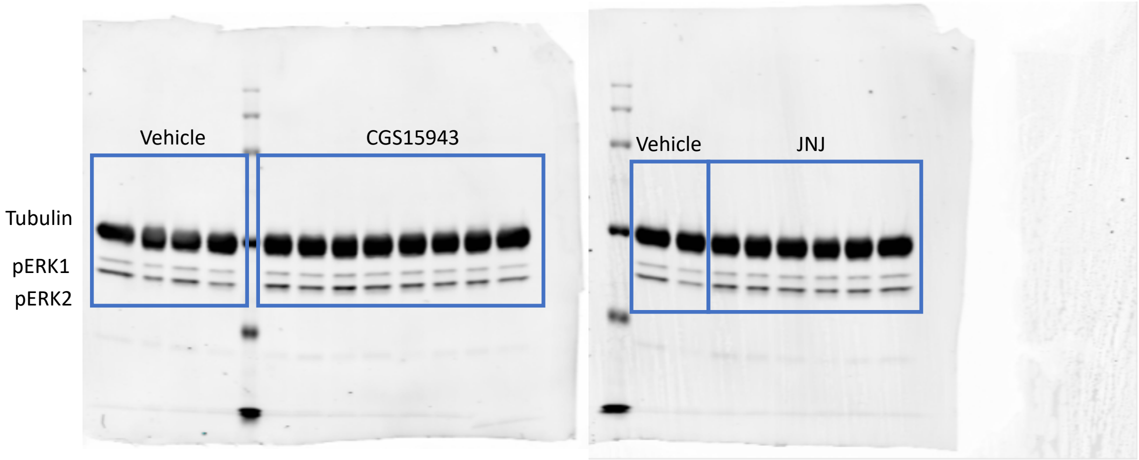

Fig 3d (pCREB)

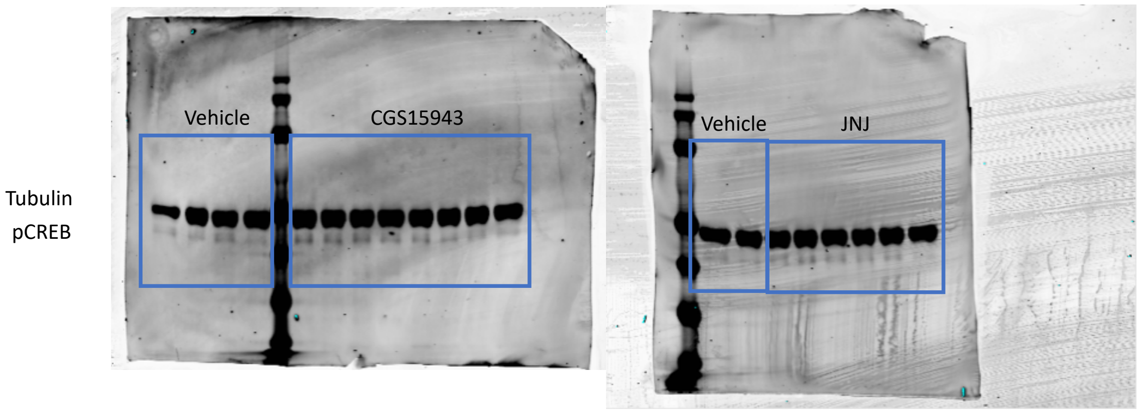

Fig. 3d (pERK) and Fig. 3d (pCREB)

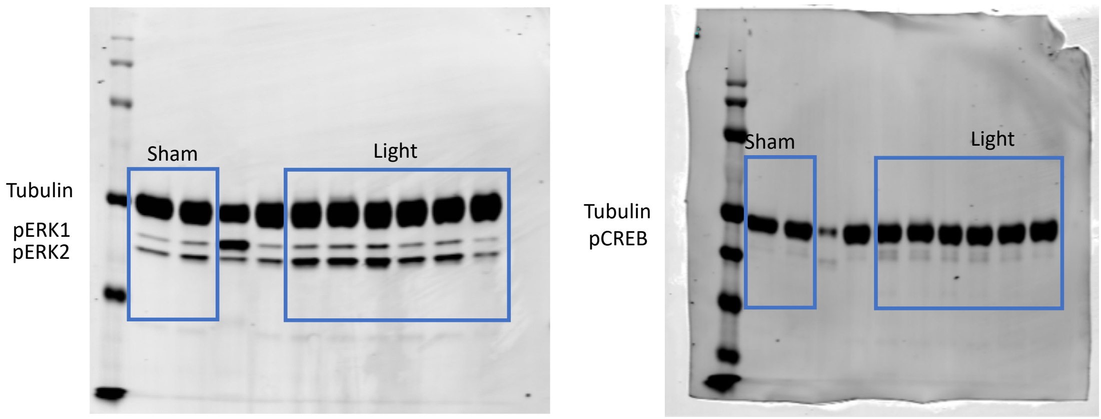

Supplement: Supplementary file 1 — Supplementary Information [file 41467_2021_22179_MOESM1_ESM.pdf]
