# Supplementary material for: Adenosine integrates light and sleep signalling for the regulation of circadian timing in mice
Source: Nat Commun. 2021 Apr 9;12:2113. doi: 10.1038/s41467-021-22179-z (PMC8035342; doi:10.1038/s41467-021-22179-z)
Supplement: Supplementary file 3 — Description of Additional Supplementary Files [file 41467_2021_22179_MOESM3_ESM.docx]

**Description of Additional Supplementary Files**

Title: Supplementary Data 1

Description: Primer Sequences

Title: Supplementary Data 2

Description: siRNA Sequences

Title: Supplementary Data 3

Description: Chemicals

Title: Supplementary Data 4

Description: Mass Spectrometry Data

Title: Supplementary Data 5

Description: Summary table of drugs used and their affinities to the adenosine receptors
