# Supplementary material for: Adenosine integrates light and sleep signalling for the regulation of circadian timing in mice
Source: Nat Commun. 2021 Apr 9;12:2113. doi: 10.1038/s41467-021-22179-z (PMC8035342; doi:10.1038/s41467-021-22179-z)
Supplement: Supplementary file 8 — Reporting Summary [file 41467_2021_22179_MOESM8_ESM.pdf]

nature research

Corresponding author(s): Aarti Jagannath
Russell Foster
Sridhar Vasudevan

Last updated by author(s): Jun 10, 2019

Reporting Summary

Nature Research wishes to improve the reproducibility of the work that we publish. This form provides structure for consistency and transparency in reporting. For further information on Nature Research policies, see Authors & Referees and the Editorial Policy Checklist .

Statistics

For all statistical analyses, confirm that the following items are present in the figure legend, table legend, main text, or Methods section.

n/a	Confirmed	
☐	☒	The exact sample size (n) for each experimental group/condition, given as a discrete number and unit of measurement
☐	☒	A statement on whether measurements were taken from distinct samples or whether the same sample was measured repeatedly
☐	☒	The statistical test(s) used AND whether they are one- or two-sided<br>*Only common tests should be described solely by name; describe more complex techniques in the Methods section.*
☒	☐	A description of all covariates tested
☐	☒	A description of any assumptions or corrections, such as tests of normality and adjustment for multiple comparisons
☐	☒	A full description of the statistical parameters including central tendency (e.g. means) or other basic estimates (e.g. regression coefficient) AND variation (e.g. standard deviation) or associated estimates of uncertainty (e.g. confidence intervals)
☒	☐	For null hypothesis testing, the test statistic (e.g. F, t, r) with confidence intervals, effect sizes, degrees of freedom and P value noted<br>*Give P values as exact values whenever suitable.*
☒	☐	For Bayesian analysis, information on the choice of priors and Markov chain Monte Carlo settings
☒	☐	For hierarchical and complex designs, identification of the appropriate level for tests and full reporting of outcomes
☒	☐	Estimates of effect sizes (e.g. Cohen's d, Pearson's r), indicating how they were calculated

Our web collection on statistics for biologists contains articles on many of the points above.

Software and code

Policy information about availability of computer code

Data collection	Actimetrics Clocklab ver 5, Multicycle 1.505; OMEGA Plate reader software, BMG Ver 5.10 R2
Data analysis	Graphpad Prism 5.0; https://github.com/randogp/STARprom

For manuscripts utilizing custom algorithms or software that are central to the research but not yet described in published literature, software must be made available to editors/reviewers. We strongly encourage code deposition in a community repository (e.g. GitHub). See the Nature Research guidelines for submitting code & software for further information.

Data

Policy information about availability of data

All manuscripts must include a data availability statement. This statement should provide the following information, where applicable:

- Accession codes, unique identifiers, or web links for publicly available datasets
- A list of figures that have associated raw data
- A description of any restrictions on data availability

All data presented as overlaid on bar charts or as box and whisker plots. Raw data (e.g. actograms) available from corresponding author upon reasonable request. Availability of code has been reported in the section 'Code Availability'.

Field-specific reporting

Please select the one below that is the best fit for your research. If you are not sure, read the appropriate sections before making your selection.

☒ Life sciences ☐ Behavioural & social sciences ☐ Ecological, evolutionary & environmental sciences

For a reference copy of the document with all sections, see nature.com/documents/nr-reporting-summary-flat.pdf

Life sciences study design

All studies must disclose on these points even when the disclosure is negative.

Sample size	Sample sizes chosen based on previous work using similar assays (e.g. circadian wheel running behaviour, luminometry) conducted within our lab. Jagannath et al., Cell, 2013.
Data exclusions	Outliers excluded from in vivo data based on abnormal behavioural patterns (e.g. abnormal wheel running bouts due to cage change etc.). These exclusion criteria was pre-specified in Figure 4 & 5.
Replication	All experiments were replicated at least twice.
Randomization	Treatment groups randomised or where the same animals received multiple treatments, treatments were rotated between trials.
Blinding	Investigators blinded during analysis

Reporting for specific materials, systems and methods

We require information from authors about some types of materials, experimental systems and methods used in many studies. Here, indicate whether each material, system or method listed is relevant to your study. If you are not sure if a list item applies to your research, read the appropriate section before selecting a response.

Materials & experimental systems

n/a	Involved in the study	
☐	☒	Antibodies
☐	☒	Eukaryotic cell lines
☒	☐	Palaeontology
☐	☒	Animals and other organisms
☒	☐	Human research participants
☒	☐	Clinical data

Methods

n/a	Involved in the study	
☒	☐	ChIP-seq
☒	☐	Flow cytometry
☒	☐	MRI-based neuroimaging

Antibodies

Antibodies used	CREB (Rabbit anti- CREB 48H2 #9197 Cell Signalling Technology, pCREB (Rabbit anti- Phospho-CREB Ser133 87G3 #9198 Cell Signalling Technology; Rabbit polyclonal antibodies to ERK1/2 (Rabbit anti- p44/42 MAPK ERK1/2 137F5 #4695, Cell Signalling Technology pERK (Rabbit anti- p44/42 MAPK ERK1/2 T202/204, #4370 Cell Signalling Technology, and A-Tubulin (Mouse anti- A-Tubulin DM1A #3873, Cell Signalling Technology. ADORA1 (AAR-006, Alomone Labs) and ADORA2a (AAR-002, Alomone Labs)
Validation	All antibodies listed above have been validated by the suppliers with relevant images on their website. Additionally all antibodies used have been cited multiple times (>5+), listed on the manufacturers website.

Eukaryotic cell lines

Policy information about cell lines

Cell line source(s)	U2OS cells - ATCC, Per2-Luc U2OS - Prof. Patrick Nolan.
Authentication	None of the cell lines were authenticated
Mycoplasma contamination	Cell lines were tested for mycoplasma and once instance of contamination was treated.
Commonly misidentified lines (See ICLAC register)	No common mis-identifed cell lines used.

Animals and other organisms

Policy information about studies involving animals; ARRIVE guidelines recommended for reporting animal research

Laboratory animals	C57Bl/6 mice, males, 80 days or over. Male mice used as female mice show scalloped circadian rhythmicity due to oestrous cycle, confounding analysis. Adk-Tg transgenic males used as above, detailed methods included. Animals housed in approximately 23 degree centigrade with around 50% humidity.

Wild animals	No wild animals used
Field-collected samples	No field collected samples used
Ethics oversight	All procedures were performed in accordance with the UK Home Office Animals (Scientific Procedures) Act 1986 and the University of Oxford's Policy on the Use of Animals in Scientific Research. (PPL 70/6382, PPL 8092CED3).

Note that full information on the approval of the study protocol must also be provided in the manuscript.
